# Impact of COVID-19 Risk Perception on Emotional Exhaustion among Chinese Hospitality Employees: The Mediating Effect of Job Insecurity

**DOI:** 10.3390/ijerph192215146

**Published:** 2022-11-17

**Authors:** Xiao Han, Mengxiao Xue, Qi Zhang, Xiaotian Dong

**Affiliations:** School of Business, Qingdao University, Qingdao 266071, China

**Keywords:** COVID-19, risk perception, job insecurity, emotional exhaustion, mindfulness, hospitality employees, conservation of resources theory

## Abstract

This study aims to investigate the levels of COVID-19 risk perception (CVRP), job insecurity (JI), and emotional exhaustion (EE) among Chinese hospitality employees to examine the mediating effect of JI on the relationship between CVRP and EE. The moderating role of employee mindfulness (MF) and perceived employability (PE) have also be examined. Data were collected from 652 hospitality employees in Shandong and Jiangsu Province, China. We used structural equation modeling (SEM) to test the hypothesized relationship among the variables. Significant relationships were found between hospitality employees’ CVRP and EE (β = 0.103, *p* < 0.01), CVRP and JI (β = 0.168, *p* < 0.001), and JI and EE (β = 0.378, *p* < 0.001). According to the results, the higher level of the CVRP of hospitality employees, the higher level of the EE. In addition, results showed mediating effects of JI on the relationship between CVRP and EE. This study also found that MF buffered the positive relationship between CVRP and EE. Therefore, in the era of COVID-19, an effective support system at the organizational level is necessary to reduce JI and EE of hospitality employees.

## 1. Introduction

The coronavirus disease 2019 (COVID-19) has become a global public health emergency that is very difficult to control. The pandemic has not only threatened people’s lives and health but also caused huge psychological pressure on the public and various psychological problems [1]. At the same time, declining operating efficiency has aggravated the burden on enterprises, brought challenges to the occupational environment of employees in various industries, and raised concern over job security [2]. Studies have found that during the COVID-19 pandemic, occupational stress has increased for employees in several industries, including healthcare, education, and hospitality [3,4,5]. Moreover, the COVID-19 pandemic has affected almost all factors related to the labor market, such as unemployment [6], work–life balance [7], psychological impairment [8], and salary reduction [9].

In China, the imposition of strict COVID-19 prevention and control measures has greatly restricted the movement of people, thus negatively affecting the hospitality industry mainly including hotels and restaurants. At the same time, frontline hospitality employees frequently come into contact with people from different regions [10], which greatly increases their risk of contracting COVID-19 [11]. In all workplaces open to the public, employees continually face the health and safety risks associated with the epidemic crisis [12]. Hospitality employees in China are defined as a high exposure risk group and need to undergo highly frequent COVID-19 nucleic acid testing. As the COVID-19 epidemic continues, strong uncertainty has begun to emerge among hospitality employees [13]. Risk perception of COVID-19 leads employees to fear losing their jobs, suffering income reduction, or losing career-development opportunities [14]. In turn, such job insecurity could lead to a series of psychological problems.

One important public health concern is emotional exhaustion, which refers to the feeling that one’s emotional and material resources are overstretched and depleted [15]. Conservation of resources (COR) theory has been widely used to explore the antecedents of emotional exhaustion [16,17]. According to COR theory, people tend to acquire and maintain valuable resources [18]. The threat to these resources posed by the perceived risk of COVID-19 could leave employees emotionally exhausted [10]. In particular, when employees feel insecure at work due to the perceived risk, employees continue to make efforts in the form of emotional labor, which intensifies energy consumption and causes emotional exhaustion [16].

This study aims to provide insights into the mechanism through which emotional exhaustion is induced by COVID-19 risk perception. We constructed a framework to examine this relationship, including the mediating role of job insecurity, using survey data collected from Chinese hospitality industry employees. Another key consideration is that people with different psychological characteristics may differ greatly in their perceptions of the same event [19]. As emotional exhaustion is caused by subjectively felt stressors, its effect would be moderated by individual differences [16]. Mindfulness and perceived employability are often considerate as factors that affect the psychological state of work. Accordingly, we examine the moderating role of mindfulness between COVID-19 risk perception and emotional exhaustion. The moderating role of perceived employability between COVID-19 risk perception and job insecurity has also been verified. Our findings suggest that both COVID-19 risk perception and job insecurity are positively associated with employees’ emotional exhaustion. Moreover, job insecurity plays a mediating role between COVID-19 risk perception and emotional exhaustion, while employee mindfulness negatively moderates between COVID-19 risk perception and emotional exhaustion.

## 2. Theoretical Background

### 2.1. COVID-19 Risk Perception and Emotional Exhaustion

Uncertainty about the COVID-19 pandemic may lead to perceptions of risk. Whereas experts can scientifically evaluate risks through statistical data, the general public relies on intuition to make risk judgments, which is often referred to as risk perception [20]. Since its sudden appearance, COVID-19 has had a wide-ranging impact. For frontline hospitality employees, the pandemic has affected many aspects of their work, daily life, and interpersonal communication, in turn, affecting their COVID-19 risk perception [21].

Emotional exhaustion is a state of fatigue resulting from excessive use of mental and emotional resources, and can result in a response to stressors in the workplace [22]. The concept is widely studied among frontline service workers [23]. Studies have shown that emotional exhaustion has a great impact on service organizations, with high levels of emotional exhaustion leading to decreased job satisfaction, organizational commitment, and job performance; lowering of employees’ self-esteem; higher incidence of temper tantrums; inhibited development of interpersonal relationships, and reduced job dedication [24].

The COVID-19 risk perception among hospitality workers may generate negative emotions such as fear, anxiety, and tension, and even lead to mental health problems such as depression [25,26,27]. Research shows that for frontline hospitality employees, perceived employment instability and reduced income and employment quality due to the COVID-19 pandemic may reduce job satisfaction, leading to a series of psychological problems and even increased turnover intention [25]. According to COR theory [28], when employees perceive the threat of resource loss, emotional exhaustion will occur. For frontline hospitality employees, whose industry has been experiencing a downturn owing to the COVID-19 pandemic, efforts devoted to stabilizing their current occupations are depleting their material and psychological resources [24]. With the persistence of the COVID-19 pandemic and strict control measures in response, Chinese frontline hospitality employees are likely to perceive consequences such as reduced income, unstable working conditions, and threats to their own health. This perception will induce higher work stress, in turn, leading to emotional exhaustion [29]. Thus, employees with high (vs. low) COVID-19 risk perception are more likely to develop emotional exhaustion. We therefore propose:

**Hypothesis 1.**  *COVID-19 risk perception among hospitality employees is positively associated with emotional exhaustion.*

### 2.2. COVID-19 Risk Perception and Job Insecurity

Job insecurity is the subjectively perceived threat to the continuity and stability of one’s employment [30,31]. Studies have shown that job insecurity is affected by organizational changes, economic fluctuations, and industry recessions [32,33]. Perceived aspects such as career-development status, working conditions, and salary levels can all impact on job insecurity [31]. According to COR theory, when resources are threatened by perceived industry risks or other factors, potential consequences may include insufficient returns on resource investment (such as not getting a good job after investing in education) and loss of existing resources (such as through unemployment) [34]. When employees lack other job opportunities, they will fear layoffs even more [35]. Amid strict travel restrictions of the COVID-19 pandemic era, Chinese hospitality employees may perceive risks of future employment difficulties and inhibited career prospects, leading to job insecurity. We thus propose:

**Hypothesis 2.**  *COVID-19 risk perception among hospitality employees is positively associated with job insecurity.*

### 2.3. The Mediating Role of Job Insecurity

As one of the most important work stressors, job insecurity entails a threat to employees’ resources, whether through imminent unemployment or reduced income [36]. Employees facing this kind of threat will experience psychological tension, the main manifestation of which is emotional exhaustion [37]. At work, employees will evaluate their investment in and returns from the organization. When their returns are lower than they perceive to be deserved, employees will be strongly motivated to protect their own resources. However, employees’ great efforts to maintain their existing jobs will bring them pressure. COR theory posits that employees who perceive job insecurity will continue to make efforts in the form of emotional labor, leading to the loss of resources and further aggravating the exhaustion of existing resources [38,39].

In a previous study, employees exposed to the threat of unemployment exhibited higher levels of negative emotions, such as stress and exhaustion, than employees not exposed to this risk [40]. Among employees, job insecurity can lead to loss of resources, exhaustion, burnout, and reduced well-being [41]. During the COVID-19 pandemic, the job insecurity experienced by hospitality employees can potentially deplete their resources and exacerbate emotional exhaustion. Arguably, therefore, COVID-19 risk perception could indirectly affect frontline employees’ emotional exhaustion through job insecurity. We thus propose:

**Hypothesis 3.**  *Job insecurity among hospitality employees is positively associated with emotional exhaustion.*

**Hypothesis 4.**  *Job insecurity mediates the relationship between hospitality employees’ COVID-19 risk perception and their emotional exhaustion.*

### 2.4. The Moderating Role of Mindfulness

Mindfulness refers to receptive attention and awareness to current events and experiences [42]. It functions as a kind of self-regulation resource. Due to differences in personal characteristics, individual mindfulness levels are also different [43]. Studies have shown that mindfulness can enhance employees’ ability to regulate emotions, promote awareness of well-being, and help alleviate negative emotions [44,45,46,47]. Mindfulness can also help employees maintain high engagement and efficiency in work, and is a skill for coping with work stress [48]. Specifically, mindfulness can reduce the uncertainty and anxiety caused by stress, and relieve employees’ emotional exhaustion [47,49]. Regarding COVID-19 risk perception, high-mindfulness employees are committed to solving current events through effective self-regulation, and could be more likely to face the emotional exhaustion caused by risks at work in a positive state [42]. Consequently, they are better able to cope with emotional exhaustion from risk perception relative to low-mindfulness employees. We thus propose:

**Hypothesis 5.**  *Mindfulness moderates the positive relationship between hospitality employees’ COVID-19 risk perception and emotional exhaustion. Specifically, the relationship is weaker for employees with high mindfulness than for employees with low mindfulness.*

### 2.5. The Moderating Role of Perceived Employability

Employability is divided into two dimensions: internal employability, which reflects the individual value of the internal labor market, and external employability, which reflects the individual value of the external labor market [50]. Studies have shown that perceived employability is a positive psychological resource for employees to control their career development, and can buffer against job insecurity [51,52,53]. Empirical findings demonstrate that employees with higher perceived employability experience higher positive emotions and greater job satisfaction [54,55]. For employees in the same work situation, those with higher perceived employability have lower job insecurity [56]. Moreover, employees with higher perceived employability are more confident in their own career development, which should reduce job uncertainties caused by the COVID-19 pandemic. When employees believe they can maintain current employment or secure future employment, the job insecurity caused by various risks can be alleviated [52]. Therefore, perceived employability may weaken the positive association between COVID-19 risk perception and job insecurity. We thus propose:

**Hypothesis 6.**  *Perceived employability moderates the positive relationship between COVID-19 risk perception and job insecurity. Specifically, this relationship is weaker for employees with high perceived employability than for employees with low perceived employability.*

**Figure 1:**
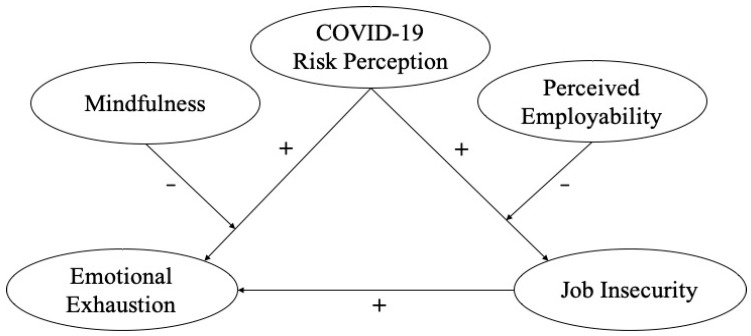
The theoretical model and hypotheses.

The theoretical model hypothesized is depicted in Figure 1.

## 3. Materials and Methods

### 3.1. Participants and Procedure

This study focused on frontline employees of the hospitality industry using a survey strategy of collecting cross-sectional data through online questionnaires. A purposive sampling technique was applied with the online questionnaire distributed to hospitality employees in organizations located in Shandong and Jiangsu provinces of China. The research sample was obtained with the help of the enterprise administrators. Data collection was conducted anonymously from July to September 2022 in the aftermath of the COVID-19 pandemic. The questionnaire consisted of questions and scales related to demographic data (gender, age, household registration, educational background, years of work, and monthly income level), COVID-19 risk perception, emotional exhaustion, job insecurity, mindfulness, and perceived employability. The multi-item scale was evaluated using a 5-point Likert scale. The link to access the online questionnaire was distributed to 652 employees, and a total of 576 respondents completed the questionnaire, with an effective recovery rate of 88.34%.

### 3.2. Measures

#### 3.2.1. COVID-19 Risk Perception

In the existing research, there are two types of scales to measure the disease-related risk perception: one is focused on health risk perception [57], and the other is mostly focus on situational awareness [58]. According to the interview with the hotel managers, we found that since the infection rate of COVID-19 in China is still low, the perceived health threat is not serious enough. In this case, we mainly considered the risk perception related to the COVID-19 situation other than health concerns. Therefore, we used an adapted scale on the basis of combining scales developed by Yoo et al. [58] and Chen and Eyoun [59]. The Cronbach’s Alpha coefficients in this study was 0.783. Sample items include: “The problem of COVID-19 is serious to Chinese Society”, “It is likely that my industry would be affected by COVID-19”, and “I am worried that I would be affected by COVID-19”. Five-point Likert scale ranging from 1 (highly disagree) to 5 (highly agree) was utilized to evaluate the items. The higher the score, the higher the COVID-19 risk perception.

#### 3.2.2. Emotional Exhaustion

Emotional exhaustion was measured using the emotional exhaustion scale from the Maslach Burnout Inventory [60]. The scale in this research included five items (e.g., “I feel used up at the end of the workday”, “I feel fatigued when I get up in the morning and have to face another day on the job”, “I feel burned out from my work”). Items were rated on a 5-point Likert scale ranging from 1 (never) to 5 (always). A higher score indicated higher emotional exhaustion. The Cronbach’s α was 0.928 in this study.

#### 3.2.3. Job Insecurity

Job insecurity was measured with a 5-item scale developed by Hellgren et al. (e.g., “I am worried about having to leave my job before I would like to”, “I feel uneasy about losing my job in the near future”, “My future career opportunities in the organization are favorable”) [61]. Five-point Likert scale ranging from 1 (highly disagree) to 5 (highly agree) was utilized to evaluate these items, and a higher score indicated higher job insecurity. The Cronbach’s α was 0.817 in the current study.

#### 3.2.4. Mindfulness

Mindfulness was measured using the mindfulness scale from the mindful attention awareness scale (MAAS) [45]. Five items from the scale were used, for example, “I break or spill things because of carelessness, not paying attention, or thinking of something else”, “I find it difficult to stay focused on what’s happening in the present”, “I find myself preoccupied with the future or the past”. Five-point Likert scale ranging from 1 (never) to 5 (always) was applied, and a higher score indicated higher mindfulness. The Cronbach’s α was 0.882 in the current study.

#### 3.2.5. Perceived Employability

Perceived employability was measured with a scale developed by Bothwell et al. [49] and the Cronbach’s α coefficient in this study was 0.855. Sample items include: “Even if there was downsizing in this organization, I am confident that I would be retained”, “My personal networks in this organization help me in my career”. This scale used a 5-point Likert scale ranging from 1 (highly disagree) to 5 (highly agree), and a higher score represented higher perceived employability.

All measurement items in the questionnaire have been shown in Table A1 in Appendix A.

### 3.3. Data Analysis

All statistical analyses were performed using SPSS 23 and PLS-SEM 4.0 software for statistical computing. We used exploratory factor analysis (EFA) to evaluate the liability and validity of the whole questionnaire. Descriptive statistics were used to examine sample demographic characteristics of participants, including gender, age, household registration, educational background, years of work, and monthly income level. Participants’ COVID-19 risk perception, emotional exhaustion, job insecurity, mindfulness, and perceived employability were then analyzed for reliability and hypothesis testing.

The KMO of this questionnaire was 0.904, where the value greater than 0.70 indicated a higher likelihood of factor analysis. The χ^2^ of Bartlett’s sphericity test was 8938.824 (*p* < 0.001). Factor analysis based on principal components extracted common factors for each dimension and orthogonal rotation was performed by varimax procedure, and the cumulative explained variance was 68.020%. The average loading values of corresponding items and corresponding dimensions for COVID-19 risk perception, emotional exhaustion, job insecurity, mindfulness, and perceived employability scales were 0.638, 0.702, 0.725, 0.680, and 0.640, respectively. Thus, the construct validity of the questionnaire was good. The values of composite reliability and Cronbach’s alpha coefficients of all factors surpass the recommended threshold of 0.70, indicating that the questionnaire had acceptable internal consistency reliability.

## 4. Results

### 4.1. Descriptive Statistics

Table 1 presents the individual socio-demographic characteristics of the 576 front-line hospitality employees in this study. Of the 576 participants, 261 were male (45.3%) and 315 were female (54.7%). The largest proportion of participants was between the ages of 20 to 39, accounting for 69.4%. In terms of educational background, 24.9% had a bachelor’s degree or above, and 75.1% had a senior school degree or below. As for years of work, 64.4% of the respondents have been in the industry for less than six years, and the remaining 35.6% have been in the industry for more than six years. More than 70% of those surveyed have a monthly income of RMB 3000 to 9000.

Table 2 reported means, standard deviations, and two-tailed zero-order correlations of the variables. Each construct’s correlations with the other constructs were exceeded by the square root of its average variance extracted, which verifies the discriminant validity of this study. The values of composite reliability (CR) of all latent variables surpass the recommended threshold of 0.80. Thus, the model has good reliability and validity.

### 4.2. Measurement Model Analysis

Table 3 indicates an acceptable model fit. As shown in Table 3, the results demonstrate that the theorized model provided a fair fit to the data. The goodness of fit indices were as follows: χ^2^ = 385.911; df = 99; χ^2^/df = 3.898, *p* < 0.001; Comparative fit index (CFI) = 0.948; Normed fit index (NFI) = 0.932; Tucker–Lewis index (TLI) = 0.938; Incremental fit index (IFI) = 0.949, Goodness of fit index (GFI) = 0.920, Adjusted goodness of fit index (AGFI) = 0.889; Standardized root mean square residual (SRMR) = 0.072; Root mean squared error of approximation (RMSEA) = 0.071. Therefore, the common method bias is not present in data.

### 4.3. Hypothesis Tests

**Figure 2:**
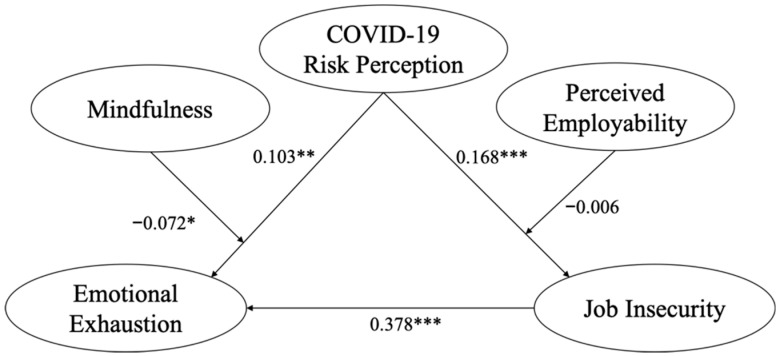
Structural model with standardized effects. Note. * *p* < 0.05, ** *p* < 0.01, *** *p* <0.001.

Structural Equation Models (SEM) were established to examine the relationship between the five variables. We fitted the data and the theoretical model by generalized least squares and modified the theoretical model based on the model fit indices. The final output model is shown in Figure 2, displaying the correlations and effect paths of the variables.

Table 4 reported the direct and indirect paths of the model. As shown in Figure 2 and Table 4, the direct effect from COVID-19 risk perception on emotional exhaustion was positive and significant (β = 0.103, *p* < 0.01), supporting Hypothesis 1. The relationship between COVID-19 risk perception and job insecurity was positive and significant (β = 0.168, *p* < 0.001). Thus, Hypothesis 2 was supported. Additionally, job insecurity was found to be significantly positively associated with emotional exhaustion (β = 0.378, *p* < 0.001), supporting Hypothesis 3. The result further showed that the indirect effect of COVID-19 risk perception on emotional exhaustion via job insecurity was significant (β = 0.063, CI [0.035, 0.094]), that is, job insecurity partially mediated the relationship between COVID-19 risk perception on emotional exhaustion, which is consistent with Hypothesis 4.

**Figure 3:**
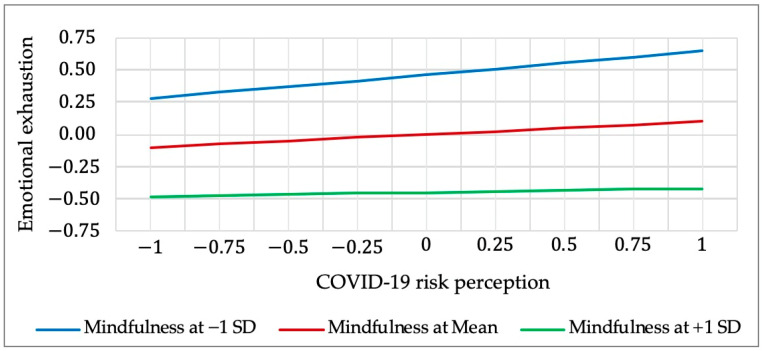
Moderating effect of mindfulness on the relationship between COVID-19 risk perception and emotional exhaustion.

In addition, the interaction role of mindfulness between COVID-19 risk perception and emotional exhaustion was negative and significant (β = −0.072, *p* < 0.05). Figure 3 shows the simple slope test results. As indicated in Figure 3, employee mindfulness buffered the positive association between COVID-19 risk perception and emotional exhaustion. The buffering effect of employees with higher level mindfulness (1 SD above the mean) was greater than those with lower level mindfulness (1 SD below the mean). Thus, Hypothesis 5 was supported. However, the moderating effect of employability between COVID-19 risk perception and job insecurity was not significant, therefore Hypothesis 6 was not supported.

## 5. Discussion and Implications

This study aimed to explore the relationship between COVID-19 risk perception, job insecurity, and emotional exhaustion among hospitality employees. Our findings confirm that COVID-19 risk perception is positively associated with emotional exhaustion, which is consistent with previous studies [59]. According to COR theory, emotional exhaustion may develop when individuals experience resource loss and feel they lack sufficient resources to cope with work stress [62]. Devoting resources to dealing with their COVID-19 risk perception depletes the resources available to hospitality employees, leaving them less able to deal with work stress, in turn, leading to emotional exhaustion.

Our study found that hospitality employees’ COVID-19 risk perception was positively associated with job insecurity, and that job insecurity mediated the positive relationship between COVID-19 risk perception and emotional exhaustion. The perceived risk of COVID-19 will cause hospitality employees to worry about the uncertain prospects for their industry and own positions, leading to job insecurity. Consistent with previous research [63,64], job insecurity will lead to increased emotional exhaustion as employees fear the threat of resource loss. The COVID-19 pandemic has caused an economic downturn and high unemployment around the world. The complex situation poses considerable uncertainty for the short-term and long-term development of the hospitality industry. Moreover, China has imposed tighter mobility restrictions on the public relative to other countries, which is especially disadvantageous for hospitality enterprises. The risks posed by these uncertainties can make hospitality employees feel less in control of their jobs, leading to job insecurity and emotional exhaustion [65].

Our research has made theoretical contributions in relevant fields. This paper has broadened the application scope of COR theory with revealing the mechanism between Chinese hospitality employees’ COVID-19 risk perception and their psychological wellbeing. Prior studies have also explained the impact of the COVID-19 epidemic on mental health in different groups. Most studies showed that perceived factors of COVID-19 would have a negative impact on mental health, which is consistent with our research. However, for different groups, the impact mechanism of COVID-19 on mental health is also different. For example, for groups with a high professional threshold such as doctors and nurses, in the COVID-19 context, burnout or anxiety is mainly caused by the increase of work intensity and disease risk [66,67,68]. In this case, job insecurity is not a major factor. This is probably because even in the face of public crisis, their strong work irreplaceability leads to less job insecurity issues. Another focus of research is migrant workers. Since the nature of their work is flexible and unstable, their fear about the COVID-19 epidemic mainly comes from their own travel restrictions or regional discrimination, which leads to negative emotions [69,70]. For this group, the sense of job insecurity under the epidemic situation is also rarely mentioned. As far as this article is concerned, the hospitality industry belongs to an industry with a low professional threshold, and the profit level of the hospitality industry is greatly affected by the flow of people. The COVID-19 risk will be directly related to the operation of the entire industry. In the case of poor operation, the possibility of its employees being laid off will increase accordingly [24,61]. Therefore, hospitality employees’ risk perception of COVID-19 will lead to emotional exhaustion through the role of job insecurity. This reveals that the psychological reactions of different groups are very different in the face of a public health crisis. In relevant psychological research, the characteristics of occupational groups should be taken into account to portray a more accurate theoretical model.

In addition, this study found that employees’ mindfulness moderated the positive relationship between COVID-19 risk perception and emotional exhaustion: specifically, this relationship was weaker among hospitality employees with higher levels of mindfulness. It can be explained that mindfulness can reduce the level of emotional exhaustion by lowering the perceived threat of resource loss. This result echoes the earlier finding that employee mindfulness helps reduce negative emotions at work [71]. Hospitality employees with higher mindfulness can focus on current matters without being overly concerned about the future, enabling them to better deal with the impact on emotional exhaustion. It should be noted, however, that one recent study among U.S. restaurant employees found no significant moderating effect of mindfulness on fear of COVID-19 and emotional exhaustion [61]. This inconsistency with our findings could be explained by differences between industries and countries.

Contrary to our expectation, perceived employability did not moderate the relationship between COVID-19 risk perception and job insecurity. Although perceived employability can help reduce hospitality employees’ fears of job losses due to the epidemic, the current downturn throughout the hospitality industry has greatly limited their career development. Even for employees with strong work competences, it is difficult to avoid the impact of the economic environment and COVID-19 restriction measures on the industry’s employment levels. These circumstances plausibly explain why our hypothesis was not supported.

Based on our findings, this study also has implications for practice. As the COVID-19 pandemic continues, hospitality enterprises should continue monitoring employees’ job insecurity and resulting emotional exhaustion, and give employees more support to meet ongoing and future challenges. In a broader sense, even if there is no longer a COVID-19 pandemic, the risks employees perceived when facing other public crises or social environment changes have a similar influence mechanism on their mental health. Some suggestions are as follows. First, hospitality managers could provide opportunities and channels for frontline employees to anonymously or publicly express negative emotions about work stress and follow up with them to provide organizational support. Second, hospitality enterprises could establish an employee assistance plan and a psychological counseling and consulting mechanism for employees, aiming to build their resilience and confidence to cope with work pressure, relieve their psychological anxiety, and offer timely assistance before emotional exhaustion. Third, organizations could provide a range of training to equip their employees with diverse skills, enabling their deployment in various positions and thereby reducing their job insecurity when facing a crisis. Finally, companies could provide training on mindfulness, hold psychology seminars, and add relevant mindfulness courses to employee wellness programs, aiming to buffer against emotional exhaustion caused by job insecurity during the COVID-19 era and beyond.

## 6. Limitations and Further Research

This study has several limitations. First, our sample included only Chinese hospitality industry employees, so it is uncertain whether our research results are generalizable to other countries or industries. Future research can further examine the impact of COVID-19 risk perception on emotional exhaustion in countries with different pandemic policies or in different industries. Second, due to the extensive understanding of COVID-19 risk perception, the scale we used may not be accurate enough. Future research can develop more appropriate scales under certain circumstances. Third, there are still many factors influencing job insecurity and emotional exhaustion that have not been explored under the COVID-19 epidemic. Future research can verify more key factors such as policy and institutional factors. Finally, because this study used cross-sectional data, causal relationships between variables cannot be established. Longitudinal or experimental designs can be conducted in future to reveal the relationships between these variables more clearly.

## 7. Conclusions

Using COR theory, this study examined the relationship between COVID-19 risk perception, job insecurity, and emotional exhaustion among hospitality employees. We found that COVID-19 risk perception was directly positively related to emotional exhaustion, as well as indirectly related to emotional exhaustion through job insecurity. Our findings also indicate that employee mindfulness can effectively alleviate the effect of job insecurity on emotional exhaustion during the COVID-19 era. This study makes theoretical contributions by demonstrating how job insecurity mediates between COVID-19 risk perception and emotional exhaustion, and by introducing the moderating effect of mindfulness. Our findings also have practical implications for hospitality industry practitioners and leaders as the pandemic continues. Future studies could generalize our findings by using data from different contexts and industries or by employing different methods to validate the model.

## Figures and Tables

**Table 1:** Demographic characteristics of participants (N = 576).

Socio-Demographic Information	N	%
**Gender**		
Male	261	45.3
Female	315	54.7
**Age, group**		
<20	47	8.2
20~29	223	38.7
30~39	177	30.7
40~49	72	12.5
50~59	51	8.9
>60	6	1.0
**Household registration**		
Local	312	54.2
Foreign provinces	264	45.8
**Educational background**		
Lower than senior school	176	30.5
Junior college	257	44.6
Bachelor degree	134	23.3
Master degree or above	9	1.6
**Years of work, group**		
<3	251	43.6
3~6	120	20.8
6~9	75	13.0
9~12	69	12.0
>12	61	10.6
**Monthly income level, group**		
<3000	81	14.1
3000~5000	222	38.5
5000~7000	101	17.5
7000~9000	83	14.4
>9000	89	15.5

**Table 2:** Descriptive statistics and correlations among variables (N = 576).

Items	M	SD	1	2	3	4	The Square Root of AVE
1. CVRP	4.229	1.051					0.770
2. EE	2.464	1.261	0.246 **				0.882
3. JI	2.734	1.226	0.174 ***	0.550 ***			0.735
4. MF	3.880	1.118	−0.195	−0.626 ***	−0.311		0.860
5. PE	3.755	1.026	−0.014	−0.321	−0.583 ***	0.201	0.794

**Note.** CVRP = COVID-19 risk perception, EE = emotional exhaustion, JI = job insecurity, MF = mindfulness, PE = perceived employability, ** *p* < 0.01, *** *p* < 0.001.

**Table 3:** Model fitting results.

Fitting	CFI	NFI	TLI	IFI	GFI	AGFI	SRMR	RMSEA
Research value	0.948	0.932	0.938	0.949	0.920	0.889	0.072	0.071

**Table 4:** Path analysis results (N = 576).

Model Pathways	Estimated	95% CI
COVID-19 risk perception → Emotional exhaustion	0.103	[0.041, 0.172]
COVID-19 risk perception → Job insecurity	0.168	[0.094, 0.232]
Job insecurity → Emotional exhaustion	0.378	[0.303, 0.454]
COVID-19 risk perception → Job insecurity → Emotional exhaustion	0.063	[0.035, 0.094]

## Data Availability

The data presented in this study are available upon request from the corresponding author. The data are not publicly available due to privacy issues.

## Appendix A

**Table A1:** Measurement items in the questionnaire.

Variables	Measurement Items
COVID-19 risk perception	The problem of COVID-19 is serious to Chinese society.
	It is likely that my industry would be affected by COVID-19. I am worried that I would be affected by COVID-19.
	I feel COVID-19 will continue in the next few years. When watching news about COVID-19 on social media, I became nervous and anxious.
Emotional exhaustion	I feel used up at the end of the workday.
	I feel fatigued when I get up in the morning and have to face another day on the job.
	I feel burned out from my work.
	I feel emotionally drained from my work.
	I feel I am working too hard on my job.
Job insecurity	I am worried about having to leave my job before I would like to.
	I feel uneasy about losing my job in the near future.
	My future career opportunities in the organization are favourable.
	There is a risk that I will have to leave my present job in the year to come.
	I feel that the organization can provide me with a stimulating job content in the near future.
	I believe that the organization will need my competence also in the future.
	My pay development in this organization is promising.
Mindfulness	I break or spill things because of carelessness, not paying attention, or thinking of something else.
	I find it difficult to stay focused on what’s happening in the present.
	I find myself preoccupied with the future or the past.
	I forget a person’s name almost as soon as I’ve been told it for the first time.
	I do jobs or tasks automatically, without being aware of what I’m doing.
Perceived employability	Even if there was downsizing in this organization, I am confident that I would be retained.
	My personal networks in this organization help me in my career.
	Among the people who do the same job as me, I am well respected in this organization.
	The skills I have gained in my present job are transferable to other occupations outside this organization.
	I could easily retrain to make myself more employable elsewhere.
